# Detection of Increased Activity of Human Parvovirus B19 Using Commercial Laboratory Testing of Clinical Samples and Source Plasma Donor Pools — United States, 2024

**DOI:** 10.15585/mmwr.mm7347a2

**Published:** 2024-11-28

**Authors:** David Alfego, Alfonso C. Hernandez-Romieu, Melissa Briggs-Hagen, Stephanie Dietz, Laura Gillim, Suzanne E. Dale, Ajay Grover, Jeffrey Albrecht, Deborah Sesok-Pizzini, Marcia Eisenberg, Cria O. Gregory, Brian Poirier

**Affiliations:** ^1^Labcorp, Burlington, North Carolina; ^2^Coronavirus and Other Respiratory Viruses Division, National Center for Immunization and Respiratory Diseases, CDC; ^3^Detect and Monitor Division, Office of Public Health Data, Surveillance, and Technology, CDC.

**Summary**

**What is already known about this topic?**

Human parvovirus B19 (B19) is a seasonal respiratory virus that causes mild disease in most persons; severe outcomes can occur in persons who are pregnant, immunocompromised, or have chronic hemolytic disorders.

**What is added by this report?**

Although no routine B19 surveillance exists in the United States, in 2024, a large U.S. commercial laboratory observed increases in percentages of positive B19 test results in clinical specimens and pooled donor source plasma compared with levels during 2018–2019.

**What are the implications for public health practice?**

Health care providers should be aware of increased B19 activity, consider testing persons at high risk for adverse B19-related outcomes, and monitor high-risk patients for complications.

## Abstract

In most persons, human parvovirus B19 (B19) causes a mild respiratory illness, but infection can result in adverse health outcomes in persons who are pregnant, immunocompromised, or who have chronic hemolytic blood disorders. During the first quarter of 2024, several European countries reported increases in B19 activity. In the United States, there is no routine surveillance for B19. To assess increases in B19 activity in the United States, trends in testing and results from two independent populations were examined: 1) the presence of immunoglobulin (Ig) M antibodies, a marker of recent infection, in clinical specimens ordered by physicians and 2) B19 nucleic acid amplification testing (NAAT) in pooled donor source plasma from a large commercial laboratory during 2018–2024. The proportion of IgM-positive clinical specimens reached 9.9% in the second quarter (Q2) of 2024 after remaining <1.5% during 2020–2023 and was higher than Q2 peaks in 2018 (3.8%, p<0.001) and 2019 (5.1%, p<0.001). The prevalence of B19–NAAT-positive donor pools (512 donations per pool) reached 20% in June 2024 after remaining <2% during 2020–2023 and was higher than peaks in 2018 (6.7%, p<0.001) and 2019 (7.3%, p<0.001). Considering the B19 activity increase in the United States in 2024, promotion of measures to prevent respiratory viruses and monitor for adverse B19-related outcomes by health care providers and public health authorities might reduce adverse health outcomes in pregnant persons and others at increased risk.

## Introduction

Human parvovirus B19 (B19) is a seasonal virus primarily transmitted through the air.^†^ B19 infection can be transmitted from a mother to the fetus during pregnancy and, rarely, through transfusion of blood components and certain plasma derivates. Most persons become infected with B19 during their school years. Immunity from infection is thought to be lifelong. The population prevalence of protective antibodies increases with age from 50% at age 20 years to approximately 70% by age 40 years (*1*).

In immunocompetent children and adults, B19 infection typically causes a mild respiratory illness. B19 can cause transient aplastic anemia (low hemoglobin with decreased reticulocytes) in persons who have chronic hemolytic blood disorders (e.g., sickle cell disease, thalassemia, and hereditary spherocytosis), and persistent aplastic anemia in persons with certain immunocompromising conditions, such as hematologic malignancies (*2*). Acute infection during pregnancy can result in infection of the fetus, which can lead to fetal anemia, hydrops fetalis, and pregnancy loss in 5%–10% of cases (*3*). No vaccine or antiviral treatment for B19 infection exists.

In the first quarter of 2024, public health authorities in several European countries observed a significant increase in cases of B19 (*4*). Because there is no routine surveillance for B19 in the United States, to determine whether B19 activity increased in 2024, the prevalences of immunoglobulin (Ig) M antibody against B19 in clinical specimens submitted to a commercial laboratory^§^ and detection of B19 through nucleic acid amplification testing (NAAT) from pooled donor source plasma were examined.

## Methods

### Data Sources

Serum B19-specific IgM antibody,^¶^ a marker of recent infection, was assessed among adults aged ≥18 years and children and adolescents aged <18 years tested at Labcorp by physician request during January 1, 2018–August 31, 2024. Routine B19 screening using NAAT of pooled donor source plasma performed at Labcorp during the same period was also analyzed.**

### Data Analysis

The number of clinical specimens tested for B19-specific IgM antibody and the proportion of positive test results were summarized monthly or quarterly by age, sex, and geographic region. Clinical specimen diagnostic test and pooled donor source plasma positivity rates were calculated as the total number of specimens considered positive divided by the number of specimens tested and the proportions of pooled specimens (512 donations per pool) with B19 detected above a 1,000 IU/mL threshold, respectively. Although the age and sex of plasma donors are unknown, historically, donor pool demographics have skewed toward males aged 20–29 years (*5*).

Peaks in positive B19 IgM and NAAT results during 2018–2019 (pre–COVID-19 pandemic), 2020–2023 (during the pandemic), and 2024 (postpandemic) were compared using Pearson’s chi-square tests, and testing volumes were compared using t-tests; a two-sided p-value <0.05 was considered statistically significant. Analyses were performed in Python (Python Software Foundation; version 3.7) using the SciPy package (version 1.14.0). This activity was reviewed by CDC, deemed not research, and was conducted consistent with applicable federal law and CDC policy.^††^

## Results

### B19 Diagnostic Testing

A total of 399,098 clinical specimens from 359,445 persons were tested for IgM antibodies during the study period. Among all clinical specimens, 369,536 (92.6%) were from adults (Supplementary Table, https://stacks.cdc.gov/view/cdc/170364); among these, 323,933 (87.7%) specimens were from women. Among specimens from children and adolescents, 15,115 (51.1%) were from females. There was no significant change in the number of specimens tested during each 6-month period between prepandemic and postpandemic periods (p = 0.05).

Among adults, the percentage of tests conducted by age group remained similar over the entire study period, with patients aged 30–39 years representing an average of 43.0% of total tests per quarter. Among children and adolescents, the highest and lowest prevalences of testing were among those aged 15–17 years (24%) and 0–2 years (9%), respectively. Children and adolescents aged 3–14 years accounted for 14%–18% of quarterly tests (Supplementary Figure 1, https://stacks.cdc.gov/view/cdc/170365).

### IgM-Positive B19 Test Results

**FIGURE 1:**
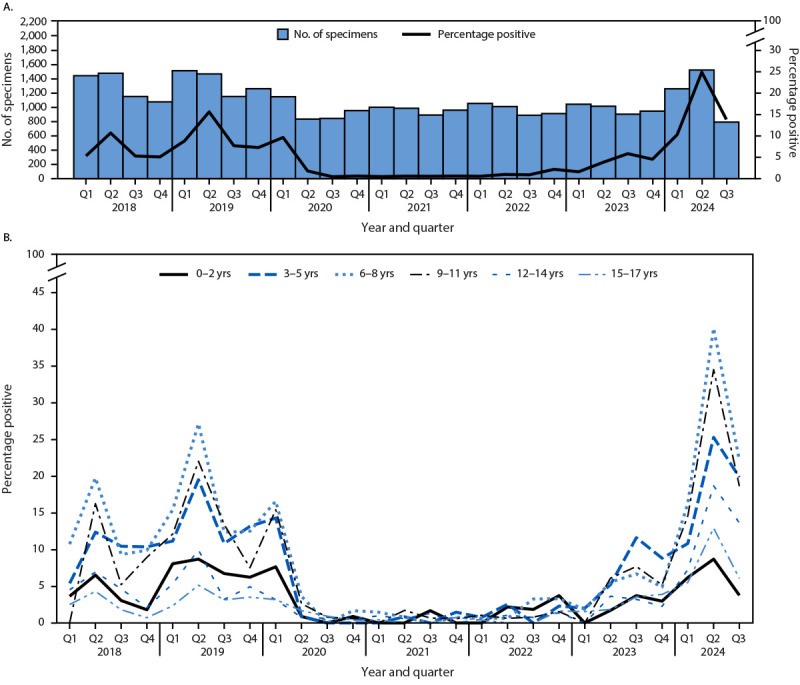
Number of clinical Parvovirus B19 specimens tested for immunoglobulin M and percentage of positive test results among children and adolescents aged <18 years, by quarter (A) and percentage of positive test results, by age group and quarter (B) — United States, 2018–2024*^,^^†^ **Abbreviation:** Q = quarter. * Q1 = January–March; Q2 = April–June; Q3 = July–September; Q4 = October–December. **^†^** Data for 2024 are through August 31.

**FIGURE 2:**
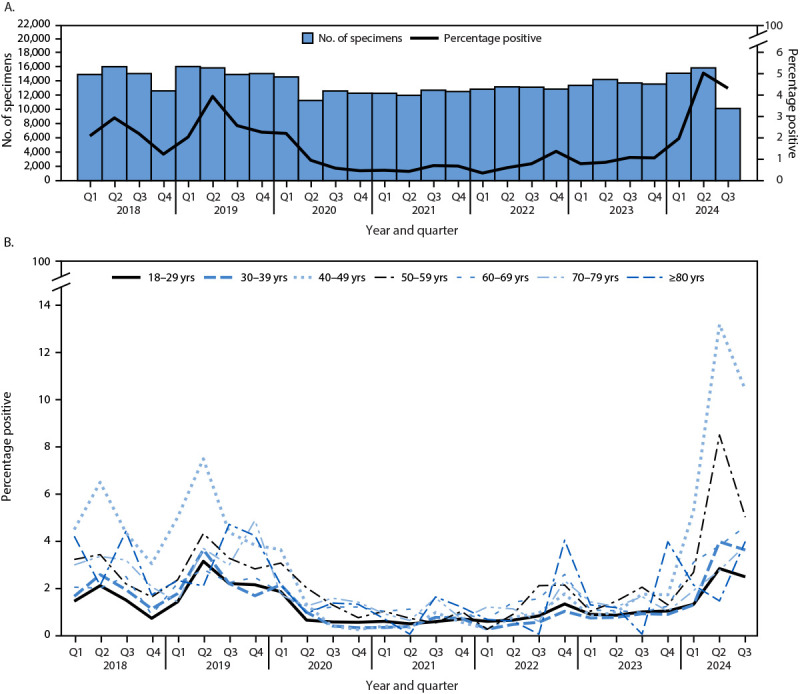
Number of clinical Parvovirus B19 specimens tested for immunoglobulin M and percentage of positive test results among adults aged ≥18 years, by quarter (A), and percentage of positive test results by age group and quarter (B) — United States, 2018–2024*^,^^†^ **Abbreviation:** Q = quarter. * Q1 = January–March; Q2 = April–June; Q3 = July–September; Q4 = October–December. **^†^** Data for 2024 are through August 31.

B19 infections, as detected by serum IgM antibodies, followed seasonal trends. In 2018 and 2019, the percentage of IgM-positive B19 test results peaked in the second quarter (Q2) of the year (April–June) for both children and adolescents (10.7% and 15.6%, respectively) (Figure 1) and adults (2.9% and 4.0%, respectively) (Figure 2). The prevalence of IgM-positive B19 test results in 2018–2019 reflects usual B19 detection in the United States. During 2020–2022, IgM seroprevalence remained low (<2%) among all age groups. The percentage of IgM-positive B19 test results began increasing from pandemic levels during the second half of 2023, and by Q2 of 2024, the percentages of IgM-positive B19 test results among children and adolescents (24.9%) and adults (5.1%) were significantly higher than were Q2 peaks during 2018 and 2019 (p<0.001 for both age groups for both years). The highest percentages of IgM-positive B19 test results in 2024 were among children aged 6–8 years (39.9% in Q2), followed by those aged 9–11 years (34.3% in Q2) (Figure 1). Among adults, the highest percentage of IgM-positive B19 results was observed in those aged 40–49 years (12.9% in Q2 2024) (Figure 2). Increases in the percentage of IgM-positive B19 results in 2024 compared with 2018–2019 were observed in all U.S. Department of Health and Human Services regions,^§§^ with the highest proportion of positive tests in Q2 of 2024 observed in regions 5 (Illinois, Indiana, Michigan, Minnesota, Ohio, and Wisconsin [11.2%]), 6 (Arkansas, Louisiana, New Mexico, Oklahoma, and Texas [10.0%]), 8 (Colorado, Montana, North Dakota, South Dakota, Utah, and Wyoming [9.1%]), and 10 (Alaska, Idaho, Oregon, and Washington [10.4%]) (Supplementary Table, https://stacks.cdc.gov/view/cdc/170364) (Supplementary Figure 2, https://stacks.cdc.gov/view/cdc/170366). As of August 31, 2024, the peak in IgM-positive B19 test results was declining as in previous seasonal cycles.

### Donor Source Plasma Pool Testing

**FIGURE 3:**
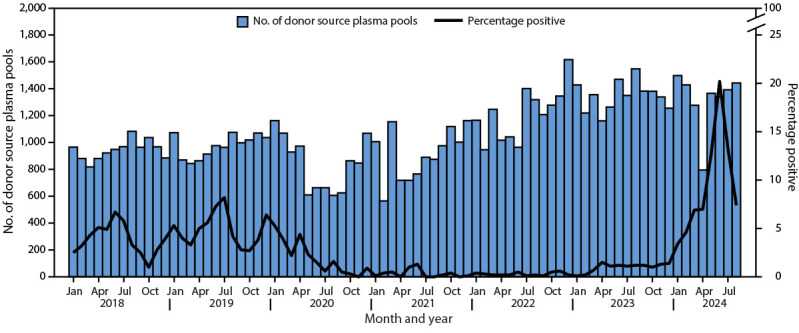
Number of donor source plasma pools* tested for parvovirus B19 and percentage of pools positive^†^ by nucleic acid amplification testing — United States, 2018–2024^§^ * The number of individual donations per pool was 512. ^†^ B19 DNA detected above a 1,000 IU/mL threshold was considered positive. ^§^ Data for 2024 are through August 31.

Donor source plasma pools, collected and tested for B19 using NAAT during the same period, followed similar trends to those observed in clinical specimen testing. A mean of 1,059 donor pools per month (SD = 246) were tested during the study period. The proportions of B19-DNA NAAT test results above a threshold of 1,000 IU/mL were elevated in July 2018 (7%) and July 2019 (8%), diminished during 2020–2023 (<2%), and reached their highest levels in June 2024 (20%) (Figure 3).

## Discussion

During the period before the COVID-19 pandemic, typical B19 transmission patterns were observed, with expected peaks in the percentage of IgM-positive clinical specimens and B19 NAAT–positive source plasma donor pools in Q2 of each year. During the COVID-19 pandemic, B19 testing of clinical specimens and source plasma donor pools decreased, and very low percentages of tests were positive. In 2024, the number of clinical specimens and source plasma donor pools tested for B19 returned to prepandemic levels, but the percentage of positive test results was higher compared with that during seasonal peaks observed during the prepandemic period. The increased percentage of positive tests in 2024 compared with that during prepandemic periods was observed in two independent populations (patients being tested for B19 by health care providers and healthy plasma donors) suggesting increased community transmission of B19 in 2024. This increase in community transmission is likely due to decreased B19 transmission during the COVID-19 pandemic, resulting from COVID-19 mitigation measures, leading to higher numbers of persons susceptible to B19 (*6*).

Patterns of transmission in 2024 were similar to those observed in prepandemic years. Peaks in the percentages of positive test results occurred in Q2 of the year, and the highest percentages of positive test results were among children aged 6–11 years. The percentage of IgM-positive B19 test results in most adult age groups in 2024 returned to prepandemic levels; however, compared with previous years, the percentage of positive B19 tests in adults aged 40–59 years was higher in 2024 than in previous years.

Health care providers, public health authorities, and the public should be aware of the likely increased circulation of B19 in the United States. CDC continues to examine syndromic surveillance and electronic health care databases to assess increases in B19 complications or adverse outcomes among groups at higher risk.

### Limitations

The findings in this report are subject to at least four limitations. First, Labcorp represents only one large laboratory network, and data are not deduplicated to the patient level. The extent to which changes in testing volume might be due to changes in laboratory market share or physicians’ test-ordering practices could not be determined, although the percentage of positive test results should not be substantially affected. Second, the demographic data available with clinical specimens is limited and precluded assessment of differences by race or ethnicity. Third, testing of clinical specimens depends on access to health care, provider recognition of potential B19 infection, and concern about development of severe B19 complications, and might therefore only represent the higher-risk or more severe B19 infections, underestimating the increase in B19 transmission and infection in the broader community. Although testing of source plasma donor pools corroborates increased community transmission, donors are not representative of the U.S. population. Finally, because positive test results could not be linked to clinical outcomes, this analysis could not assess a change in complications or adverse outcomes among groups at higher risk, including persons who are pregnant, immunocompromised, or who have chronic hemolytic anemias.

### Implications for Public Health Practice

CDC released a Health Advisory on August 13, 2024, with recommendations for health care providers, health departments, and the public (*7*). Although B19 causes mild disease in most persons, those who are pregnant, who have certain immunocompromising conditions (e.g., leukemia or other cancers, organ transplant, HIV infection, or current chemotherapy), or who have chronic hemolytic blood disorders (e.g., sickle cell disease, thalassemia, or hereditary spherocytosis) are at increased risk for severe complications from B19 infection. Health care providers should have an increased index of suspicion for B19 among persons evaluated with fever, rash, arthropathy, or unexplained anemia with low reticulocyte count and should consider B19 testing for persons at increased risk for severe complications, including pregnant persons who might have been exposed to B19. Some European countries have observed a significant increase in fetal morbidity and mortality several months after increased community transmission of B19 (*8*). Health care providers caring for pregnant persons should be particularly vigilant for signs of reduced fetal movement or evidence of hydrops which could be associated with B19 (*9*). Transmission of B19 in the community and school setting can be mitigated by promoting prevention strategies against respiratory illness, such as taking steps for cleaner air (e.g., facilitating circulation of fresh outside air, purifying indoor air, or gathering outdoors), practicing good hygiene, masking, and following guidance for kindergarten through 12th grade schools (*10*). Persons at high risk for severe B19 complications who work or study in settings with elevated risk for B19 exposure should consider additional prevention strategies, such as masking, to reduce their risk for infection.

## Footnotes

* These authors contributed equally to this report.

† In the United States, B19 transmission generally occurs during late winter and early spring.

§
https://www.labcorp.com/

¶ IgM antibodies were assayed using a Food and Drug Administration (FDA)–cleared enzyme immunoassay. An index value of >1.1 indicates antibody detection. Isolated whole blood B19 NAAT tests accounted for 4.1% of all diagnostic testing and were not included in the analysis.

** Since 2009, FDA has recommended testing all plasma-derived products and plasma units for parvovirus B19 using nucleic acid tests. Whole blood is not screened for parvovirus B19 in the United States. https://www.fda.gov/regulatory-information/search-fda-guidance-documents/nucleic-acid-testing-reduce-possible-risk-parvovirus-b19-transmission-plasma-derived-products

†† 45 C.F.R. part 46, 21 C.F.R. part 56; 42 U.S.C. Sect. 241(d); 5 U.S.C. Sect. 552a; 44 U.S.C. Sect. 3501 et seq.

§§
https://www.hhs.gov/about/agencies/iea/regional-offices/index.html
